# The predicative value of early quantitative electroencephalograph in epilepsy after severe traumatic brain injury in children

**DOI:** 10.3389/fped.2024.1370692

**Published:** 2024-08-15

**Authors:** Wei Bai

**Affiliations:** Department of Pediatrics, Xiangyang NO.1 People’s Hospital, Xiangyang, Hubei, China

**Keywords:** early quantitative electroencephalograph (EEG), severe traumatic brain injury (TBI) in children, post-traumatic epilepsy (PTE), peak envelope analysis, epilepsy

## Abstract

**Objective:**

To explore whether early quantitative electroencephalograph (EEG) can predict the development of epilepsy in pediatric patients with severe traumatic brain injury (TBI).

**Methods:**

A total of 78 children with severe TBI who were admitted to our hospital were divided into post-traumatic epilepsy (PTE) and non-PTE groups according to whether or not they developed PTE. EEGs of frontal, central and parietal lobes were recorded at the time of their admission. The power values of each frequency band, odds ratio and peak envelope power values of each brain region were statistically analyzed. In addition, the patients were followed up for two years, and the occurrence of PTE was documented.

**Results:**

During the follow-up period, PTE occurred in 8 patients. Analysis of EEG signals across different brain regions (frontal, central, and parietal lobes) revealed significant differences between the PTE and non-PTE groups. Patients with PTE exhibited significantly higher δ and θ power values (*P* < 0.01), lower α/θ ratios (*P* < 0.01), and elevated θ/β, (δ + θ)/(α + β), and peak envelope power (*P* < 0.01) compared to those in the non-PTE group.

**Conclusion:**

In children with severe TBI, the parameter characterization of early quantitative EEG has potential application in predicting PTE.

## Introduction

Traumatic brain injury (TBI) is the leading cause of injury and death in children, caused by external forces or trauma to the brain (1). It is estimated that approximately 475,000 children aged 0–14 years suffer from TBI each year (2). They may develop a range of sequelae following a TBI (3). Post-traumatic epilepsy (PTE) is one of the most severe sequelae (4), accounting for 20% of all sequelae and 5% of all epilepsy cases (5, 6). Studies have revealed an overall incidence of PTE after pediatric TBI of 10% (7). PTE seriously affects the recovery and long-term quality of life of TBI patients (8, 9). It has been reported that the risk of PTE increases with the severity of TBI (10, 11). Therefore, early detection and treatment of PTE are important to improve treatment outcomes of TBI children.

Electroencephalogram (EEG) monitoring is a widely used clinical tool in neurosurgery. EEG can be used for the diagnosis, seizure risk assessment, and long-term prognosis prediction of epilepsy patients (12, 13). In recent years, new EEG monitoring techniques have been emerging, such as continuous electroencephalogram (CEEG) and quantitative electroencephalogram (QEEG). CEEG allows real-time assessment of cortical function in acutely ill patients and is used for monitoring epileptic seizures (14); QEEG allows feature extraction of specific parameters of the EEG signal, such as power spectra, complexity metrics, and percentage of inhibition in the frequency bands (15). QEEG can identify epileptic seizures and abnormal discharge signals from the brain (16). Santiago-Rodrıguez reported that, compared to the EEG of normal subjects, the δ, α, and β-band power values of adolescent patients with myoclonus epilepsy were increased (17).

Early research on predicting PTE with EEG did not yield the desired outcomes. One study focused on identifying abnormal EEG signals only based on gross image features found no significant association between these gross image features and the development of PTE at various time points after trauma (18). In addition, EEGs are typically obtained weeks to months after trauma, and seizures exhibit cyclical patterns, factors that undoubtedly limit the utility of EEG in predicting PTE. Moreover, the role of EEG as a monitoring treatment guideline for epilepsy remains unclear (19).

The advancement of statistical concepts and artificial intelligence algorithms has enabled clinicians to make more timely and accurate predictions and intervene early in disease diagnosis and treatment (20). These techniques have facilitated the exploration of QEEG parameters in TBI, including power values in each frequency band, odds ratio, and peak envelope power (21). In pediatric patients with PTE, there is potential to reduce epileptic symptoms through early detection and intervention. For instance, Liesemer et al. (22) suggest that early post-traumatic seizures (EPTS) occur in a significant proportion of pediatric TBI cases, especially within the first 12 h post-injury, and by identifying key risk factors such as young age, severe TBI and non-accidental trauma, they stratified patients at higher risk for EPTS and demonstrated that early intervention with antiepileptic drugs were protective against EPTS, indicating the potential for reducing epileptic symptoms through early detection and treatment. Additionally, integrating QEEG monitoring could facilitate personalized treatment strategies (23), further supporting the importance of early intervention in managing post-traumatic epilepsy in pediatric patients (24).

However, currently, there is no effective method to identify patients at high risk of PTE. Therefore, this study aimed to explore whether early EEG features can determine the occurrence of PTE in children with severe TBI. EEG data were collected from patients upon admission, and QEEG features were extracted and analyzed in various brain regions. Patients were then followed up for two years to monitor the occurrence of epilepsy. The discovery of these early electrophysiologic features of PTE will aid in the early diagnosis and treatment of TBI in children.

## Materials and methods

### Study subjects

Patients with severe TBI who were admitted to our hospital were selected for this study, following which a total of 78 children were identified as eligible for the study. The study was approved by the Ethics Committee of our hospital. Informed consent was signed by the patient's guardian, and verbal consent was obtained from the patient. All the study procedures were performed in accordance with the approved ethical guidelines and the Declaration of Helsinki.

### Inclusion and exclusion criteria

The inclusion criteria comprised patients aged between 2 months and 18 years old, diagnosed with severe TBI presenting a Glasgow Coma Scale (GCS) score of ≤8 upon admission, with a clear history of trauma, and with informed consent obtained from their guardian. Exclusion criteria comprised patients who were deceased or in imminent brain death upon admission (indicated by pupil dilation or absence of reflex to light, GCS score = 3), underwent decompressive craniectomy, those diagnosed with epilepsy before the occurrence of TBI, patients who received prophylactic antiepileptic medication, those diagnosed with idiopathic epilepsy during the follow-up period, and those deemed by neurosurgeons to have a chronic disease posing a risk of inducing epileptic seizures.

### Follow-up

We conducted a 2-year follow-up to determine the occurrence of PTE. Based on previous literature (25), PTE in this study was characterized as a singular occurrence of epilepsy manifesting beyond 7 days post-trauma (i.e., delayed). The diagnosis of epilepsy was established either based on clinical symptoms or through EEG findings. Patients were then classified into either the PTE group or the non-PTE group based on whether they developed PTE.

### Data collection

Data regarding age, gender, time elapsed from injury to hospitalization, mechanism of injury [classified as direct violence (assault, intentionally inflicted upon the individual by another person) or indirect violence (accidental injury, the injury is not directly inflicted by another person but is still a result of violent circumstances or events such as falls or road traffic incidents)], and GCS score within 24 h of admission were collected for all TBI patients enrolled in this study. Subsequently, another researcher verified the completeness and authenticity of the data at the conclusion of case data collection.

### EEG signal acquisition and analysis

EEG monitoring was performed at the time of patient admission utilizing a NicoletOne Monitor (Natus Neurology Incorporated, USA). The duration of EEG recordings varied, with most sessions lasting between one and two hours. Continuous monitoring was implemented for critically ill patients as required. The analyzed EEGs were obtained within the first 24 h of admission to capture early electrophysiological changes.

Some of the patient's hair was removed, and the scalp was sterilized with alcohol. The patient was assisted to lie flat. Scrubs were used to cover the electrode placement sites. A total of 19 electrodes were placed on the scalp in the left frontal (Fp1), right temporal (Fp2), left frontal (F3), right frontal (F4), left anterior temporal (F7), right anterior temporal (F8), left central (C3), right central (C4), left temporal (T3), right temporal (T4), left posterior temporal (T5), right posterior temporal (T6), left parietal (P3), right parietal (P4), left occipital (O1), right occipital (O2), frontal midline (Fz), central midline (Cz), and parietal midline (Pz). A conductive paste was used to increase electrical conductivity, and a headgear was utilized to secure the electrodes in place, with Cz designated as the reference electrode and the prefrontal and mid-frontal electrodes serving as grounding electrodes. Electrode impedance was maintained below 5 KΩ, and a stimulus frequency of 500 Hz was administered. Importantly, recording sessions were conducted in a quiet environment to mitigate external interference. EEG signals exhibiting obvious pseudo-errors were excluded from the analysis.

The brain was segmented into three regions: the frontal lobe (comprising F3, Fz, and F4), central lobe (C3, Cz, and C4), and parietal lobe (P3, Pz, and P4) (18, 19). Quantitative analysis of EEG characteristics from these regions was conducted. Utilizing the NicoletOne system (Natus Quantum Incorporated, USA), QEEG data were automatically extracted and integrated based on the raw EEG recordings. Amplitude-dependent EEG data were collected at an average interval of every 1 s, while frequency-dependent EEG data were sampled every 10 s. Power values corresponding to different frequency bands [δ (1–4 Hz), θ (4–8 Hz), α (8–13 Hz), and β (13–30 Hz)] were quantified from each electrode site, and ratios such as α/θ, α/β, θ/β, and (δ + θ)/(α + β) were computed.

Peak envelope analysis was used to clarify the trend of the EEG signal amplitude. Time was designated as the independent variable, and initially, the peak and trough points of the EEG amplitude within the 0–25 Hz range were identified. The envelope was then derived by connecting these points. Graphical data were transformed into quantitative values using the same method described above.

### Statistical analysis

All data were statistically analyzed using SPSS 20.0 software. Count data are expressed as rate (%), and the chi-square test was used to compare two groups. Measurement data are expressed as mean ± standard deviation (SD), and the difference between the two groups was analyzed using the *t*-test. *P* < 0.05 was considered statistically significant.

## Results

### Baseline characteristics of patients

The study included 78 children with severe TBI, among whom 8 developed PTE during the follow-up period, resulting in an incidence rate of 11.6%. Within the PTE group, there were 7 boys and 1 girls, with an average age of 12.56 ± 2.41 years, a mean time from injury to hospitalization of 3.56 ± 0.50 h, and varying causes of injury including 5 cases of direct violence, 3 cases of indirect violence. Their GCS score averaged 6.62 ± 0.70. In contrast, the non-PTE group consisted of 61 boys and 9 girls, with an average age of 12.53 ± 2.35 years, a mean time from injury to hospitalization of 3.27 ± 0.86 h, and injury causes comprising 41 cases of direct violence, 29 cases of indirect violence, and 6 cases of infection. Their GCS score averaged 6.82 ± 0.39 (Table 1). No statistically significant differences were observed between the two groups regarding baseline characteristics (*P* > 0.05).

**Table 1:** Comparison of baseline characteristics between the two groups of patients.

Baseline information	Non-PTE group (*n* = 70)	PTE group (*n* = 8)	*χ*^2^/*t*	*P*
Age (year)	12.53 ± 2.35	12.56 ± 2.41	−0.033	0.973
Gender (*n*, %)			0.001	0.977
Boy	61 (87.14)	7 (87.5)		
Girl	9 (12.86)	1 (12.5)		
Time from injury to hospitalization (h)	3.27 ± 0.86	3.56 ± 0.50	−0.906	0.368
Cause of injury (*n*, %)			0.046	0.831
Direct violence	41 (58.6)	5 (62.5)		
Indirect violence	29 (41.4)	3 (37.5)		
GCS score	6.82 ± 0.39	6.62 ± 0.70	1.235	0.221

PTE, post-traumatic epilepsy; GCS, glasgow coma scale.

### QEEG analysis of various brain regions of patients

Analysis of EEG signals across different brain regions revealed significant differences between patients in the PTE and non-PTE groups. In the frontal lobe, patients with PTE exhibited significantly higher δ and θ power values (*P* < 0.001), lower α/θ ratios (*P *= 0.018), and elevated θ/β (*P* = 0.002), (δ + θ)/(α + β), and peak envelope power (*P* < 0.001) compared to those in the non-PTE group. Similarly, within the central lobe, the PTE group demonstrated markedly higher δ and θ power values (*P* < 0.001), lower α/θ ratios (*P* < 0.001) and increased θ/β, (δ + θ)/(α + β), and peak envelope power (*P* < 0.001) relative to the non-PTE group. In the parietal lobe, patients with PTE exhibited significantly higher δ and θ power values (*P* < 0.001), lower α/θ ratios (*P* < 0.001) and higher θ/β, (δ + θ)/(α + β), and peak envelope power (*P* < 0.001) compared to the non-PTE group (Table 2). Notably, the most pronounced changes in QEEG data were observed in the frontal region.In addition, for each band, we calculate the potential threshold as the average of the mean values of the PTE and non-PTE groups. This approach offers a simple yet effective threshold for clinical differentiation.

**Table 2:** Comparison of quantitative EEG data of frontal, central and parietal brain regions in two groups of patients.

Region	EEG parameter	Non-PTE group (*n* = 70)	PTE group (*n* = 8)	Threshold (Hz)	*t*	*P*
Frontal lobe	δ	11.50 ± 1.75	16.35 ± 2.68	13.93	−6.985	<0.001
	θ	15.66 ± 2.64	20.19 ± 4.54	17.93	−4.234	<0.001
	α	40.38 ± 4.95	41.99 ± 5.46	41.19	−0.863	0.391
	β	25.21 ± 3.36	25.26 ± 3.33	25.24	−0.043	0.966
	α/θ	2.65 ± 0.56	2.15 ± 0.44		2.427	0.018
	α/β	1.72 ± 0.37	1.67 ± 0.17		0.392	0.696
	θ/β	0.63 ± 0.14	0.82 ± 0.27		−3.237	0.002
	(δ+θ)/(α+β)	0.42 ± 0.06	0.55 ± 0.07		−5.544	<0.001
	Peak envelope power	0.28 ± 0.4	0.84 ± 0.09		−33.352	<0.001
Central lobe	δ	11.22 ± 1.50	13.89 ± 1.52	12.56	−4.754	<0.001
	θ	16.86 ± 1.75	21.38 ± 1.33	19.12	−7.054	<0.001
	α	43.55 ± 4.16	43.11 ± 3.81	43.33	0.288	0.774
	β	25.60 ± 2.38	25.53 ± 1.63	25.57	0.071	0.944
	α/θ	2.61 ± 0.36	2.03 ± 0.29		4.357	<0.001
	α/β	1.78 ± 0.29	1.70 ± 0.19		0.760	0.449
	θ/β	0.66 ± 0.10	0.84 ± 0.07		−4.990	<0.001
	(δ+θ)/(α+β)	0.41 ± 0.04	0.52 ± 0.05		−6.482	<0.001
	Peak envelope power	0.42 ± 0.04	0.75 ± 0.04		−20.453	<0.001
Parietal lobe	δ	13.05 ± 1.31	17.82 ± 1.24	15.44	−9.791	<0.001
	θ	16.03 ± 2.22	21.60 ± 1.79	18.82	−6.836	<0.001
	α	46.00 ± 3.71	47.64 ± 3.26	46.82	−1.195	0.236
	β	25.79 ± 2.66	24.34 ± 3.30	25.07	1.427	0.158
	α/θ	2.93 ± 0.50	2.22 ± 0.26		3.938	<0.001
	α/β	1.92 ± 0.36	1.98 ± 0.22		−0.475	0.636
	θ/β	0.63 ± 0.10	0.90 ± 0.14		−7.060	<0.001
	(δ+θ)/(α+β)	0.41 ± 0.04	0.55 ± 0.07		−8.433	<0.001
	Peak envelope power	0.38 ± 0.05	0.81 ± 0.04		−25.352	<0.001

PTE, post-traumatic epilepsy; EEG, electroencephalograph.

## Discussion

In this study, we observed distinctive characteristics in the early QEEG data of children who developed PTE. Notably, the most pronounced changes in QEEG data were detected in the frontal lobes among PTE patients, with decreased α/θ ratio and increased peak envelope power evident across all three brain regions. Our findings suggest that QEEG analysis conducted shortly after admission can serve as a predictive tool for assessing the long-term risk of PTE in children with severe TBI (26). Early identification of epilepsy risk facilitates the establishment of an optimal window for initiating antiepileptic therapy in TBI children, as plasticity mechanisms within the central nervous system are most effective when intervened early post-injury.

A total of 8 patients developed PTE during the 2-year follow-up period, resulting in an incidence rate of 11.6%, which is lower than that reported in previous studies (27, 28). CEEG has demonstrated utility in predicting epilepsy risk within one year following acute brain injury (29). Similarly, in patients with moderate to severe TBI, various early QEEG features, including absolute power values and variability, accurately predict patient outcomes at one year (30). However, whether early EEG data correlate with the long-term risk of developing PTE remains unclear. Our study, with its extended 2-year follow-up period, provides valuable insights into this aspect, surpassing the durations of previous investigations and facilitating a more comprehensive exploration of the long-term PTE risk.

Epileptiform discharges predominantly originate from excitatory pyramidal neurons within the cortex, with their highest prevalence observed in the central, frontal, and temporal lobes among epileptic individuals (31, 32). Therefore, our study focused on analyzing QEEG data from various brain regions. We observed that in the early EEG recordings of children with TBI who later developed PTE, the abnormal parameters were predominantly localized in the frontal lobe. Specifically, we identified a total of 8 parameters significantly differing from those in the non-PTE group, which was followed by alterations observed in the parietal lobe, where 4 parameters exhibited significant changes. Conversely, the central lobe showed the least pronounced alterations, with only 3 parameters demonstrating significant differences between the PTE and non-PTE groups.

In the frontal lobe, our study identified several parameters in the PTE group that significantly differed from those in the non-PTE group, with the exception of the β power value. Additionally, we observed significant alterations in the α/θ ratio and peak envelope power across all three brain regions. These findings are consistent with a previous investigation, which reported a lower α/θ ratio in the temporal region of patients with temporal lobe epilepsy compared to controls (33). Importantly, our study represents the first application of peak envelope analysis to EEG analysis in children, offering a novel marker for predicting future PTE.

Our findings suggest that early EEG parameters hold promise in predicting PTE in children with TBI, facilitating timely interventions to mitigate epilepsy-related harm. However, PTE risk prediction should not rely solely on EEG analysis, as alternative techniques also demonstrate predictive capabilities. For instance, the combination of MRI and CEEG has shown efficacy in predicting seizures in TBI patients (34). To enhance the accuracy of long-term PTE risk prediction, we propose integrating EEG parameters with imaging data.

This study had several limitations that should be acknowledged. First, due to data limitations, including a small cohort size and a follow-up period of only 2 years, our dataset was not sufficient to accurately assess the impact of antiepileptic drug administration on PTE incidence. Furthermore, while there may be standardized thresholds for EEG monitoring in clinical practice, the specific length of monitoring may vary depending on the context and institution. Additionally, we did not have specific data on the number of patients discharged with antiepileptic drugs (AEDs). Future studies should include data related to AED use alone to better understand the role of early AED intervention in preventing PTE. Given that this study represents an initial exploration into this field, these limitations should be considered when interpreting the findings and further studies are required for validation.

## Conclusion

Our study highlights the prognostic value of QEEG in assessing PTE development in children with severe TBI. The significant differences observed in QEEG parameters between PTE and non-PTE patients underscore the potential utility of QEEG. Specifically, the frontal, central and parietal EEG parameters of PTE children changed significantly in the early stages of trauma, with the most significant changes in the frontal lobe. Moreover, the α/θ ratio and peak envelope power were significantly altered in all the three brain regions. Collectively, these findings provide insights for consideration of QEEG monitoring as part of the clinical management of pediatric TBI patients to facilitate early identification of those at risk for PTE. However, further research is warranted to validate these findings and to elucidate the specific role of QEEG in guiding therapeutic interventions and improving patient outcomes.

## Data Availability

The original contributions presented in the study are included in the article/Supplementary Material, further inquiries can be directed to the corresponding author.
